# Genes and (Common) Pathways Underlying Drug Addiction

**DOI:** 10.1371/journal.pcbi.0040002

**Published:** 2008-01-04

**Authors:** Chuan-Yun Li, Xizeng Mao, Liping Wei

**Affiliations:** Center for Bioinformatics, National Laboratory of Protein Engineering and Plant Genetic Engineering, College of Life Sciences, Peking University, Beijing, People's Republic of China; SRI Artificial Intelligence Center, United States of America

## Abstract

Drug addiction is a serious worldwide problem with strong genetic and environmental influences. Different technologies have revealed a variety of genes and pathways underlying addiction; however, each individual technology can be biased and incomplete. We integrated 2,343 items of evidence from peer-reviewed publications between 1976 and 2006 linking genes and chromosome regions to addiction by single-gene strategies, microrray, proteomics, or genetic studies. We identified 1,500 human addiction-related genes and developed KARG (http://karg.cbi.pku.edu.cn), the first molecular database for addiction-related genes with extensive annotations and a friendly Web interface. We then performed a meta-analysis of 396 genes that were supported by two or more independent items of evidence to identify 18 molecular pathways that were statistically significantly enriched, covering both upstream signaling events and downstream effects. Five molecular pathways significantly enriched for all four different types of addictive drugs were identified as common pathways which may underlie shared rewarding and addictive actions, including two new ones, GnRH signaling pathway and gap junction. We connected the common pathways into a hypothetical common molecular network for addiction. We observed that fast and slow positive feedback loops were interlinked through CAMKII, which may provide clues to explain some of the irreversible features of addiction.

## Author Summary

Drug addiction has become one of the most serious problems in the world. It has been estimated that genetic factors contribute to 40%–60% of the vulnerability to drug addiction, and environmental factors provide the remainder. What are the genes and pathways underlying addiction? Is there a common molecular network underlying addiction to different abusive substances? Is there any network property that may explain the long-lived and often irreversible molecular and structural changes after addiction? These important questions were traditionally studied experimentally. The explosion of genomic and proteomic data in recent years both enabled and necessitated bioinformatic studies of addiction. We integrated data derived from multiple technology platforms and collected 2,343 items of evidence linking genes and chromosome regions to addiction. We identified 18 statistically significantly enriched molecular pathways. In particular, five of them were common for four types of addictive drugs, which may underlie shared rewarding and addictive actions, including two new ones, GnRH signaling pathway and gap junction. We connected the common pathways into a hypothetical common molecular network for addiction. We observed that fast and slow positive feedback loops were interlinked through CAMKII, which may provide clues to explain some of the irreversible features of addiction.

## Introduction

Drug addiction, defined as “the loss of control over drug use, or the compulsive seeking and taking of drugs despite adverse consequences,” has become one of the most serious problems in the world [1]. It has been estimated that genetic factors contribute to 40%–60% of the vulnerability to drug addiction, and environmental factors provide the remainder [2]. What are the genes and pathways underlying addiction? Is there a common molecular network underlying addiction to different abusive substances? Is there any network property that may explain the long-lived and often irreversible molecular and structural changes after addiction? These are all important questions that need to be answered in order to understand and control drug addiction.

Knowing the genes and vulnerable chromosome regions that are related to addiction is an important first step. Over the past three decades, a number of technologies have been used to generate such candidate genes or vulnerable chromosome regions. For example, in hypothesis-driven studies, genes in different brain regions were selectively expressed, downregulated, or knocked out in animal models of addiction [3]. Recent high-throughput expression-profiling technologies such as microarray and proteomics analyses identified candidate genes and proteins whose expression level changed significantly among different states in addiction [4,5]. Finally, genetic studies such as animal Quantitative Trait Locus (QTL) studies, genetic linkage studies, and population association studies identified chromosomal regions that may contribute to vulnerability to addiction [6–8]. However, as addiction involves a wide range of genes and complicated mechanisms, any individual technology platform or study may be limited or biased [3,9–14]. There is a need to combine data across technology platforms and studies that may complement one another [3,15,16]. The resultant gene list, preferably in a database form with additional functional information, would be a valuable resource for the community. Systematic and statistical analysis of the genes and the underlying pathways may provide a more complete picture of the molecular mechanism underlying drug addiction.

Although different addictive drugs have disparate pharmacological effects, there are also similarities after acute and chronic exposure such as acute rewarding and negative emotional symptoms upon drug withdrawal [17]. Recently it was asked “Is there a common molecular pathway for addiction?” because elucidation of common molecular pathways underlying shared rewarding and addictive actions may help the development of effective treatments for a wide range of addictive disorders [17]. Several individual pathways have been proposed as common pathways [17]; however, they have not been studied systematically and statistically.

Key behavioral abnormalities associated with addiction are long-lived with stable and irreversible molecular and structural changes in the brain, implying a “molecular and structural switch” from controlled drug intake to compulsive drug abuse [18]. It was proposed that the progress of addiction may involve positive feedback loops that were known to make continuous processes discontinuous and reversible processes irreversible [19]. Once a common molecular network for addiction is constructed, we can look for the existence of positive feedback loops in the network and study the coupling between the loops. It may provide clues to explain the network behaviour and the addiction process.

## Results

### Most Comprehensive Collection and Database of Addiction-Related Genes to Date

**Figure 1:**
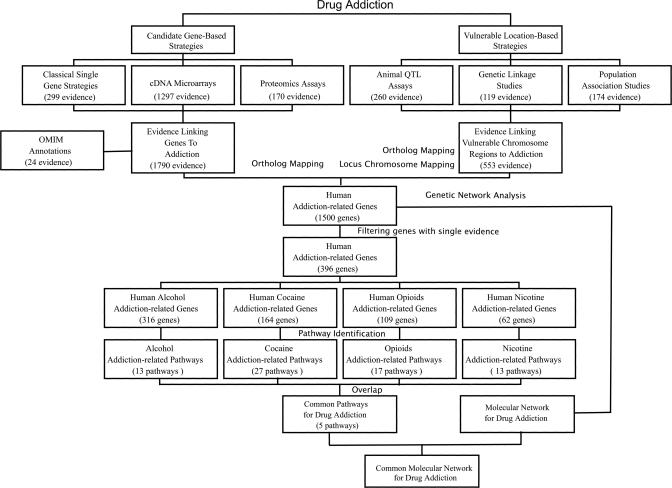
Pipeline for Collection of Data and Identification of (Common) Molecular Networks for Drug Addiction Strategies used to study the genetic and environmental influences underlying addiction were divided into two types. Candidate gene-based strategies identified a list of genes related to addiction, including candidate genes identified in classical animal models, significantly differentially expressed genes identified in microarray or proteomics assays, and OMIM annotations. Strategies focused on genetic factors identified a list of addiction-vulnerable regions through animal QTL studies, genetic linkage studies, and population association studies. We integrated these datasets and obtained a list of human addiction-related genes. This dataset was then divided into four subsets based on addictive drugs, and analyzed using KOBAS, a statistical method to identify enriched molecular pathways. Molecular pathways enriched for all subsets were considered to be common pathways for drug addiction, which were further connected to construct a common molecular network underlying different types of addiction.

As currently the information is scattered in literature, we retrieved and reviewed more than 1,000 peer-reviewed publications from between 1976 and 2006 linking genes and chromosome regions to addiction. In total, we collected 2,343 items of evidence linking 1,500 human genes to addiction. The detailed statistics is shown in Figure 1 and Table S1. A Knowledgebase of Addiction-Related Genes (KARG) is made publicly available at http://karg.cbi.pku.edu.cn. A description of the database statistics is given in Table S1, and the functional annotation fields are listed in Table S2. Two screenshots of the database user interface are shown in Figures S1 and S2. The interface supports browsing of the genes by chromosome or pathways, advanced text search by gene ID, organism, type of addictive substance, technology platform, protein domain, and/or PUBMED ID, and sequence search by BLAST similarity [20]. All data, database schema, and MySQL commands are freely available for download at http://karg.cbi.pku.edu.cn/download.php.

### Statistically Significantly Enriched Pathways in Addiction-Related Genes

We analyzed in detail 396 genes that were supported by two or more independent items of evidence. We found that 18 pathways were statistically significantly enriched in addiction-related genes compared to the whole genome as background, including both metabolic and signalling pathways (Table 1). These pathways could be clustered into two categories: (i) upstream events of drug addiction including crosstalk among MAPK signaling, insulin signalling, and calcium signalling, which share properties with long-term potentiation; and (ii) downstream effects including regulation of glycolysis metabolism, regulation of the actin cytoskeleton, and apoptosis, which share components with a list of neurodegenerative disorders such as Huntington disease and amyotrophic lateral sclerosis. Gene Ontology enrichment analysis confirmed the findings (see details in Text S1 and Table S3).

**Table 1:**
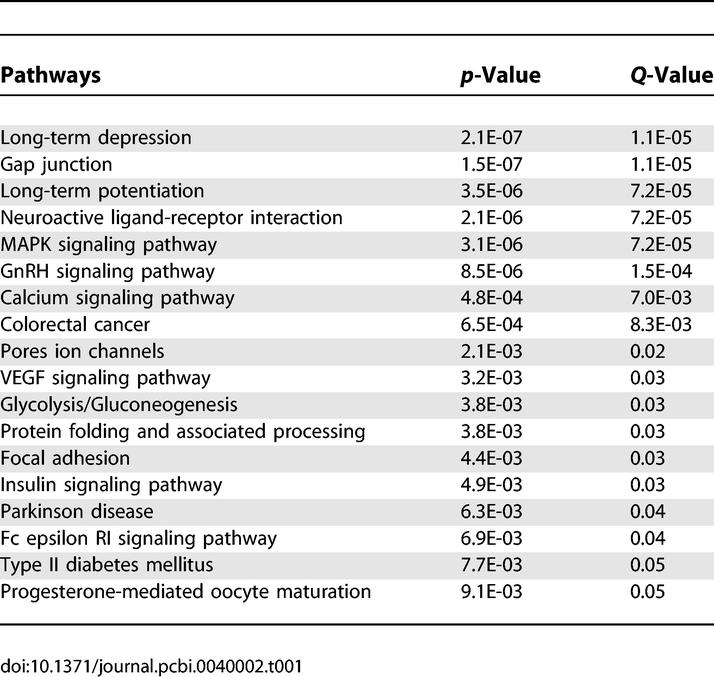
Significantly Enriched KEGG Pathways for Addiction-Related Genes

### Common Molecular Pathways for Drug Addiction

Because we collected metadata about each item of evidence linking genes to addiction, in particular the nature of the addictive substance, we could ask next what are the pathways underlying addiction to each type of substance, and what are the common pathways among them. We identified five pathways shared by all four addictive substances (Table 2). Three of the pathways had been linked to addictive behaviors in previous studies and were statistically confirmed here. For example, “long-term potentiation” had been linked to addiction-induced adaptations in glutamatergic transmission and synaptic plasticity [21]. In particular, a core component of this pathway, CAMKII, had been reported to regulate neurite extension and synapse formation through regulation of the actin cytoskeleton [22], providing possible explanations for morphological changes triggered by addictive drugs [17]. This pathway was also considered a key molecular circuit underling the memory system, highlighting the possible shared mechanisms between drug addiction and the learning and memory system [23]. “MAPK signaling pathway” is another example, as previous studies had suggested its roles in regulating synaptic plasticity related to long-lasting changes in both memory function and addictive properties [24].

**Table 2:**
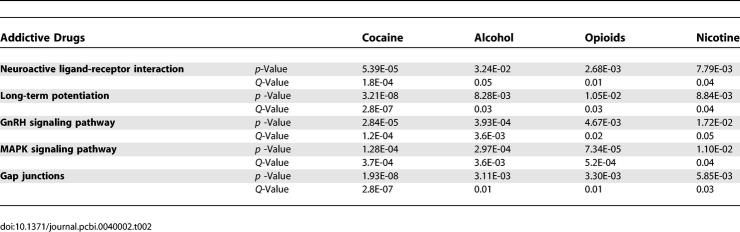
Common Molecular Pathways Identified in Different Types of Drug Addiction

More interestingly, two other common pathways identified here had not been directly linked to addiction. “GnRH signaling pathway” was reported to activate gene expression and secretion of gonadotropins and regulate stress pathways in the hypothalamo-pituitary gonadal axis and mammalian reproduction [25]. It is reasonable to hypothesize that the pathway may also be involved in the regulation and control of certain emotional behaviors in addiction such as stress-induced drug-seeking. Another common pathway identified in our study, “Gap junctions”, can be regulated directly by three addiction-related kinases in the “long-term potentiation” pathway, PKA, PKC, and ERK. Since gap junctions are not only an important type of connection for neuroglial cells but also the most prevalent group of electrical synapses in the brain [26], this regulation may imply potential modification of cell communication in addiction. It would be interesting to investigate the roles of these pathways in future experimental studies.

**Figure 2:**
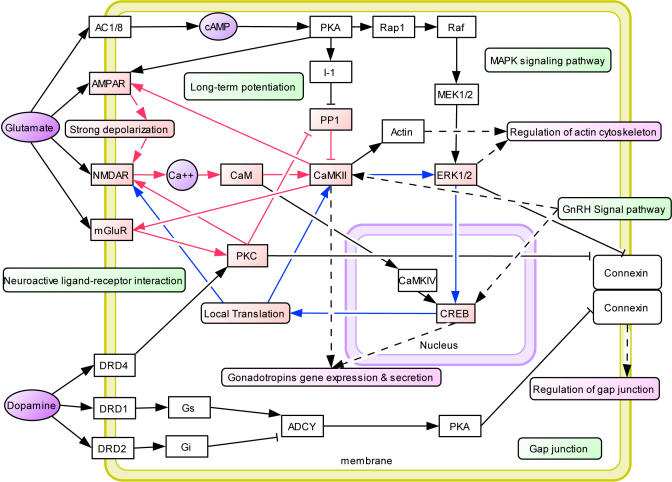
Hypothetical Common Molecular Network for Drug Addiction The network was constructed manually based on the common pathways identified in our study and protein interaction data. Addiction-related genes were represented as white boxes while neurotransmitters and secondary massagers were highlighted in purple. The common pathways are highlighted in green boxes. Related functional modules such as “regulation of cytoskeleton”, “regulation of cell cycle”, “regulation of gap junction”, and “gene expression and secretion of gonadotropins” were highlighted in carmine boxes. Several positive feedback loops were identified in this network. Fast positive feedback loops were highlighted in red lines and slow ones were highlighted in blue lines.

A pathway is in itself a subjective concept, whereas the real systems are dynamic and include wide-ranging crosstalk among functional modules. Connecting the common pathways with additional protein–protein interaction data, we constructed a hypothetical common molecular network for drug addiction, shown in Figure 2 (see details in Text S2 and Figure S3).

### Positive Feedback Loops in the Network

From the common pathway network we identified four positive feedback loops, shown in Figure 2. We further observed that they interlinked with each other through CAMKII (Figure 2). Two of these positive feedback loops involved signal transduction and would be considered “fast” loops, whereas the other two loops involved transcription and translation and would be considered “slow” loops. It had been reported in a dozen systems, such as budding yeast polarization and Xenopus oocyte maturation, that coupled fast and slow positive feedback loops could create a switch that was inducible and resistant to noise and played key roles in discontinuous and irreversible biological process, features characteristic of addiction [27–29]. It was also known that activation of CAMKII played key roles in the development and maintenance of addiction states [30,31]. Disruption of dendritic CaMKII translation impaired the stabilization of synaptic plasticity and memory consolidation [32,33]. These evidences, taken together, suggested that the fast and slow positive feedback loops interlinked through CAMKII may be essential for the development and consolidation of addiction and may provide a systems-level explanation for some of the characteristics of addictive disorders.

## Discussion

The addiction-related genes, (common) pathways, and networks were traditionally studied experimentally. The explosion of genomic and proteomic data in recent years both enabled and necessitated bioinformatic studies of addiction. Integration of data from multiple sources could remove biases of any single technology platform, and statistical and network analysis of the integrated data could uncover high-level patterns not detectable in any individual study. For instance, our analysis revealed not only many pathways already implicated in addiction [34–38], but also new ones such as GnRH signaling pathway and gap junction, as well as the coupled positive feedback loops through CAMKII. They could serve as interesting hypotheses for further experimental testing.

The collection of addiction-related genes and pathways in KARG, the first bioinformatic database for addiction, is the most comprehensive to date. However, as new technologies continue to be developed and used, more and more genes will be linked to addiction. In 2004, a paper asked why proteomics technology was not introduced to the field of drug addiction [5]; since then eleven studies have identified about 100 differentially expressed proteins in drug addiction. Tilling-array technology, another new strategy for whole-genome identification of transcription factors binding sites, has been used to identify targets of CREB, an important transcription factor implicated in drug addiction [39]. In addition, as 100 K and 500 K SNP arrays have been introduced recently, whole genome association studies will also identify more closely packed and unbiased hypothesis-free vulnerable positions [40]. We will continue to integrate new data and update the gene list and molecular pathways toward a better understanding of drug addiction.

## Materials and Methods

### Collection of addiction-related genes.

The data collection pipeline is summarized in Figure 1. The data and knowledge linking genes and chromosome regions to addiction were extracted from reviewing more than 1,000 peer-reviewed publications from between 1976 and 2006. This list of publications, available on KARG Web site at http://karg.cbi.pku.edu.cn/pmid.php, included recent review papers on addiction selected from results of PUBMED query ‘(addiction OR “drug abuse") AND review' as well as research papers selected from PUBMED query ‘(addiction OR “drug abuse") AND (gene OR microarray OR proteomics OR QTL OR “population association” OR “genetic linkage”)'. The data spanned multiple technology platforms including classical hypothesis-testing of single genes, identification of significantly differentially expressed genes in microarray experiments, identification of significantly differentially expressed proteins in proteomics assays, identification of addiction-vulnerable chromosome regions in animal QTL studies, genetic linkage studies, population association studies, and OMIM annotations [41]. From each publication we collected the genes, proteins, or chromosome regions linked to addiction, as well as metadata such as species, nature of the addictive substance, studied brain regions, technology platforms, and experimental parameters. For candidate genes or chromosomal regions identified in mouse or rat, we mapped them to human genes through ortholog mapping by Homologene or syntenic mapping, respectively [41]. For chromosome regions identified in genetic studies, we identified candidate genes when at least one positive marker lay (i) within the gene or (ii) in 3′ or 5′ flanking sequences that were contained on a block of high restricted haplotype diversity along with exon sequences from the same gene [8]. In total, we collected 2,343 items of evidence linking 1,500 human genes to addiction. Among them 396 genes were supported by two or more items of evidence (see full list in Table S4). This more reliable subset was used in subsequent analysis.

### Identification of pathways statistically significantly enriched in addiction-related genes

We used the FASTA sequences of the 396 human addiction-related genes as input to the KOBAS software, using all known genes in the human genome as background [42,43]. KOBAS had been shown to lead to experimentally validated pathways [44]. It maps the input sequences to similar sequences in known pathways in the KEGG database [45] (as determined by BLAST similarity search with evaluated cut off e-values <1e-5, rank ≤10), and then groups the input genes by pathways. Because some pathways are naturally large, they may appear highly represented in a random selection of genes or gene products. To resolve this, KOBAS selects the pathways that are more likely to be biologically meaningful by calculating the statistical significance of each pathway in the input set of genes or gene products against all pathways in the whole genome as background. For each pathway that occurs in the input genes, KOBAS counts the total number of genes in the input that are involved in the pathway, named m, and the total number of genes in the whole genome that are involved in the same pathway, named M. If input has n genes and the whole genome has N genes, the *p*-value of the pathway is calculated using a hypergeometric distribution:

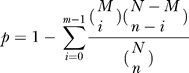



KOBAS then performs FDR correction [42] to adjust for multiple testing. Pathways with FDR-corrected *Q*-value < 0.05 were considered statistically significantly enriched in the input set of addiction-related genes.

### Identification of “common” molecular pathways and network.

For each of the four addictive substances, cocaine, opiate, alcohol and nicotine, we input its list of related genes to KOBAS to identify the statistically significantly enriched pathways. Molecular pathways that were identified as significantly enriched for all four addictive substances were selected as common pathways for drug addiction.

We constructed a large molecular network of addiction-related genes with the nodes being the gene products and the links extracted from the KEGG database, the Biomolecular Interaction Network Database (BIND), and Human Interactome Map (HIMAP) [46]. The network was analyzed and visualized by Medusa [47]. We selected a more biologically meaningful sub-network representing only the common pathways identified above.

### Development of a database for addiction-related genes.

We developed a database with MySQL relational schema. Cross-reference to key external databases were included to integrate functional information about the genes, such as gene annotation [41], Gene Ontology annotation [48], interacting proteins [46], and functional domain annotations [49]. In addition, a link was given to the original literature reference in the NCBI PubMed database [41]. We implemented a Web-based user interface of the database using PHP and queries of the database using PHP/SQL query script.

## Supporting Information

Figure S1 Chromosome View of Addiction-Related Genes and Genetic Vulnerability Points for Addiction In window (A), + and − indicate addiction-related genes on the plus or the minus chain, respectively, while ‘*' labels addiction-vulnerable points identified in population association studies. Clicking blue + or − in the (A) window links to detailed descriptions of that gene (B), including basic information, evidence implicating it in addiction and various functional annotations. Clicking the red stars links to a detailed description of this genetic vulnerable point (C), including evidence for implication in addiction and functional annotations of this point (such as the nearest genes and possible effects on these genes).
(1.4 MB TIF)

Figure S2 Pathway View of Addiction-Related Genes In window (A), statistically significant pathways are listed with *p*-values and *Q*-values. Clicking pathway names link to (B), pages showing interactive charts for that pathway, derived from KEGG. In the chart, addiction-related genes are highlighted in red. Detailed descriptions of each gene can be retrieved when clicking genes in the chart.
(932 KB TIF)

Figure S3 Protein Interaction Networks of Addiction-Related Genes On the basis of KEGG data and protein interaction data deposited in BIND and HIMAP, we developed a hypothetical addiction-related molecular network using the whole set of human addiction-related genes (A). The network was analyzed and visualized by Medusa. Upstream events, including crosstalk among the MAPK pathway, insulin signaling, and calcium signaling, are highlighted in the yellow square, while events implicated in cell development and communication are marked in red circles (including focal adhesion, adhesion junction, tight junction, gap junction, and axon guidance). Genes implicated in neurodegeneration are highlighted as diamonds. It is clear that genes in upstream events and downstream events have an interface, which are further manually separated and visualized (B). Genes represented in this interface are highlighted in purple. Genes represented in upstream events or downstream events, which have direct interaction with interface genes, are highlighted in red or blue, respectively. Especially, several genes having more than three interactions with interface genes are highlighted in green. This subnetwork may provide a screenshot to explain the relationship between upstream kinase signaling pathways and downstream events such as cytoskeletal modification.
(6.2 MB TIF)

Table S1 Statistics of the Database (34 KB DOC)

Table S2 Contents of Database Entries (36 KB DOC)

Table S3 Significantly Enriched Gene Ontology Terms in Addiction-Related Genes (73 KB DOC)

Table S4 Addiction-Related Genes for Four Addictive Substances (713 KB DOC)

Text S1 Gene Ontology Annotation and Functional Enrichment Analysis (25 KB DOC)

Text S2 Molecular Interaction Networks Underlying Drug Addiction (35 KB DOC)

## Abbreviations

KARG: Knowledgebase of Addiction-Related Genes
QTL: quantitative trait locus
