# Supplementary material for: Genes and (Common) Pathways Underlying Drug Addiction
Source: PLoS Comput Biol. 2008 Jan 4;4(1):e2. doi: 10.1371/journal.pcbi.0040002 (PMC2174978; doi:10.1371/journal.pcbi.0040002)
Supplement: Supplementary file 1 [file pcbi.0040002.sd001.doc]

**Supplementary Text S1: Gene Ontology annotation and functional enrichment analysis**

Among the1500 human addiction-related genes, 396 had two or more independent items of evidence, which were considered to be more reliable and survived the subsequent filtering process. Genes in the set of 396 were annotated by Gene Ontology (GO) (Ashburner, et al., 2000). Terms of the fifth level of GO Biological Function were used in our annotations and significantly enriched GO terms were identified using DAVID (Dennis, et al., 2003). P-values for GO terms were calculated using a hypergeometric distribution with the background of whole genome human genes, as described previously (Dennis, et al., 2003).

For addiction-related genes, a total of 36 significantly enriched terms from the fifth level of GO Biological Function were identified (Supplementary Table S3). These terms can be mainly clustered into six functional categories: carbohydrate catabolism, regulation of apoptosis and proliferation, cytoskeleton and neuronal development, neurotransmitter regulation and several upstream kinase-signaling pathways**.** These functions could be considered global properties of drug addiction.

Ashburner, M., et al. (2000) Gene ontology: tool for the unification of biology. The Gene Ontology Consortium, *Nat Genet*, **25**, 25-29.

Dennis, G., Jr., et al. (2003) DAVID: Database for Annotation, Visualization, and Integrated Discovery, *Genome Biol*, **4**, P3.
