# Supplementary material for: Genes and (Common) Pathways Underlying Drug Addiction
Source: PLoS Comput Biol. 2008 Jan 4;4(1):e2. doi: 10.1371/journal.pcbi.0040002 (PMC2174978; doi:10.1371/journal.pcbi.0040002)
Supplement: Supplementary file 2 [file pcbi.0040002.sd002.doc]

**Supplementary Text S2: Molecular Interaction Networks underlying Drug Addiction**

“Pathway” is in itself a subjective concept, since the real systems are dynamic and include wide-ranging crosstalk among functional modules. It is necessary and important to consider addiction-related genes in the context of complex molecular networks. We therefore annotated human addiction-related genes with various molecular interaction databases and analyzed the genetic interaction network (Supplementary Figure S3).  The upstream and downstream events discussed in the previous section represent the most complex part in the network. It seems that there is a complex interaction interface linking upstream and downstream events.  Genes in this interface were manually separated and visualized as a sub-network, providing a screenshot for the relationship between upstream kinase-signaling pathways and downstream effects such as cytoskeleton modifications.
