# Supplementary material for: Genes and (Common) Pathways Underlying Drug Addiction
Source: PLoS Comput Biol. 2008 Jan 4;4(1):e2. doi: 10.1371/journal.pcbi.0040002 (PMC2174978; doi:10.1371/journal.pcbi.0040002)
Supplement: Supplementary file 3 [file pcbi.0040002.st001.doc]

**Supplementary Table S1: Statistics of the database**

| **Evidence Origin** | **Evidence Type** | **Gene Count** | **Evidence Count** |
| --- | --- | --- | --- |
| **Candidate Gene-Based Strategies** | | **1221** | **1790** |
| Microarray | Genes encoding significantly differentially expressed mRNAs | 949 | 1297 |
| Proteomics | Genes encoding significantly differentially expressed proteins | 119 | 170 |
| Single gene strategies | Genes implicated in addiction | 185 | 299 |
| OMIM | Database annotations | 24 | 24 |
| **Vulnerability Location-Based Strategies** | | | **553** |
| Animal QTL | Addiction-vulnerable Regions | | 260 |
| Genetic Linkage | Addiction-vulnerable peak regions | | 119 |
| Population Association | Addiction-vulnerable points/clusters | | 174 |
