# Supplementary material for: Genes and (Common) Pathways Underlying Drug Addiction
Source: PLoS Comput Biol. 2008 Jan 4;4(1):e2. doi: 10.1371/journal.pcbi.0040002 (PMC2174978; doi:10.1371/journal.pcbi.0040002)
Supplement: Supplementary file 4 [file pcbi.0040002.st002.doc]

**Supplementary Table S2: Contents of database entries**

| **Entry Fields** | **Descriptions and Data Resources** |
| --- | --- |
| **Basic Information** | Basic information for this gene, including Gene ID, official symbol, aliases, links to OMIM, links to HPRD, chromosome and location, gene descriptions, gene type and genomic context. Data from NCBI ENTREZ GENE |
| **Evidence Summary** | A summary of evidence linking this gene to addiction |
| **KARG Evidence** | For each piece of evidence, KARG internal ID, organism, evidence ID and links, addictive drug types and addiction brain areas were annotated. For large-scale profiling evidence, differentially expressed effects and fold-changes were also included. |
| **Gene Ontology** | Gene Ontology annotations. Data from GO website |
| **Interaction information** | Protein interaction annotations. Data from BIND and HPRD |
| **Domain Information** | Protein domain annotations. Data from INTERPRO |
