# Supplementary material for: Genes and (Common) Pathways Underlying Drug Addiction
Source: PLoS Comput Biol. 2008 Jan 4;4(1):e2. doi: 10.1371/journal.pcbi.0040002 (PMC2174978; doi:10.1371/journal.pcbi.0040002)
Supplement: Supplementary file 5 [file pcbi.0040002.st003.doc]

**Supplementary Table S3: Significantly enriched Gene Ontology terms in addiction-related genes**

| **Terms** | **P-Value** |
| --- | --- |
| synaptic transmission | 1.25E-20 |
| neuron differentiation | 5.27E-09 |
| regulation of action potential | 1.82E-07 |
| second-messenger-mediated signaling | 7.21E-07 |
| regulation of apoptosis | 2.09E-06 |
| regulation of programmed cell death | 2.32E-06 |
| apoptosis | 4.00E-06 |
| negative regulation of programmed cell death | 5.17E-06 |
| cation homeostasis | 2.08E-05 |
| neuron maturation | 3.35E-05 |
| negative regulation of protein kinase activity | 3.39E-04 |
| glutamate signaling pathway | 8.03E-04 |
| regulation of protein kinase activity | 8.07E-04 |
| phosphate metabolism | 9.95E-04 |
| cellular morphogenesis during differentiation | 1.40E-03 |
| response to reactive oxygen species | 1.80E-03 |
| alcohol biosynthesis | 2.20E-03 |
| enzyme linked receptor protein signaling pathway | 3.62E-03 |
| G-protein coupled receptor protein signaling pathway | 4.35E-03 |
| neuron morphogenesis during differentiation | 4.44E-03 |
| cell migration | 5.53E-03 |
| regulation of neurogenesis | 6.20E-03 |
| carbohydrate catabolism | 6.64E-03 |
| negative regulation of cell proliferation | 7.05E-03 |
| monoamine transport | 9.35E-03 |
| response to oxidative stress | 1.26E-02 |
| oxidative phosphorylation | 1.52E-02 |
| alcohol catabolism | 1.66E-02 |
| chemotaxis | 1.66E-02 |
| neurotransmitter transport | 2.46E-02 |
| positive regulation of programmed cell death | 2.87E-02 |
| ion transport | 3.14E-02 |
| monosaccharide metabolism | 3.17E-02 |
| carboxylic acid metabolism | 3.72E-02 |
| regulation of progression through cell cycle | 3.74E-02 |
| axon guidance | 4.90E-02 |
