# Supplementary material for: Genes and (Common) Pathways Underlying Drug Addiction
Source: PLoS Comput Biol. 2008 Jan 4;4(1):e2. doi: 10.1371/journal.pcbi.0040002 (PMC2174978; doi:10.1371/journal.pcbi.0040002)
Supplement: Supplementary file 6 [file pcbi.0040002.st004.doc]

**Supplementary Table S4: Addiction-related genes for four addictive substances**

| **Entrez Gene ID** | **Official Symbol** | **Locations** | **Gene description** | Cocaine | **Alcohol** | **Opioids** | **Nicotine** |
| --- | --- | --- | --- | --- | --- | --- | --- |
| 12 | SERPINA3 | 14q32.1 | serpin peptidase inhibitor, clade A (alpha-1 antiproteinase, antitrypsin), member 3 | Yes | Yes | Yes | Yes |
| 18 | ABAT | 16p13.2 | 4-aminobutyrate aminotransferase | Yes | Yes | - | Yes |
| 88 | ACTN2 | 1q42-q43 | actinin, alpha 2 | Yes | Yes | Yes | Yes |
| 112 | ADCY6 | 12q12-q13 | adenylate cyclase 6 | - | - | Yes | - |
| 114 | ADCY8 | 8q24 | adenylate cyclase 8 (brain) | Yes | Yes | Yes | Yes |
| 143 | PARP4 | 13q11 | poly (ADP-ribose) polymerase family, member 4 | - | Yes | - | - |
| 213 | ALB | 4q11-q13 | albumin | Yes | - | Yes | - |
| 230 | ALDOC | 17cen-q12 | aldolase C, fructose-bisphosphate | - | Yes | Yes | - |
| 231 | AKR1B1 | 7q35 | aldo-keto reductase family 1, member B1 (aldose reductase) | Yes | Yes | Yes | Yes |
| 242 | ALOX12B | 17p13.1 | arachidonate 12-lipoxygenase, 12R type | - | Yes | - | - |
| 347 | APOD | 3q26.2-qter | apolipoprotein D | - | Yes | - | - |
| 348 | APOE | 19q13.2 | apolipoprotein E | Yes | Yes | - | - |
| 351 | APP | 21q21.2\|21q21.3 | amyloid beta (A4) precursor protein (peptidase nexin-II, Alzheimer disease) | Yes | Yes | - | - |
| 358 | AQP1 | 7p14 | aquaporin 1 (Colton blood group) | - | Yes | - | - |
| 419 | ART3 | 4p15.1-p14 | ADP-ribosyltransferase 3 | - | Yes | - | - |
| 427 | ASAH1 | 8p22-p21.3 | N-acylsphingosine amidohydrolase (acid ceramidase) 1 | Yes | Yes | - | - |
| 478 | ATP1A3 | 19q13.31 | ATPase, Na+/K+ transporting, alpha 3 polypeptide | Yes | - | - | - |
| 506 | ATP5B | 12q13.13 | ATP synthase, H+ transporting, mitochondrial F1 complex, beta polypeptide | Yes | - | Yes | - |
| 526 | ATP6V1B2 | 8p22-p21 | ATPase, H+ transporting, lysosomal 56/58kDa, V1 subunit B2 | - | Yes | Yes | - |
| 529 | ATP6V1E1 | 22pter-q11.2\|22q11.1 | ATPase, H+ transporting, lysosomal 31kDa, V1 subunit E1 | - | Yes | Yes | - |
| 595 | CCND1 | 11q13 | cyclin D1 | - | Yes | - | - |
| 598 | BCL2L1 | 20q11.21 | BCL2-like 1 | Yes | - | - | - |
| 627 | BDNF | 11p13 | brain-derived neurotrophic factor | Yes | Yes | Yes | Yes |
| 687 | KLF9 | 9q13 | Kruppel-like factor 9 | - | Yes | - | - |
| 725 | C4BPB | 1q32 | complement component 4 binding protein, beta | Yes | Yes | Yes | Yes |
| 760 | CA2 | 8q22 | carbonic anhydrase II | - | Yes | - | - |
| 776 | CACNA1D | 3p14.3 | calcium channel, voltage-dependent, L type, alpha 1D subunit | - | Yes | - | - |
| 786 | CACNG1 | 17q24 | calcium channel, voltage-dependent, gamma subunit 1 | - | Yes | - | - |
| 794 | CALB2 | 16q22.2 | calbindin 2, 29kDa (calretinin) | - | Yes | - | - |
| 796 | CALCA | 11p15.2-p15.1 | calcitonin/calcitonin-related polypeptide, alpha | - | Yes | - | - |
| 805 | CALM2 | 2p21 | calmodulin 2 (phosphorylase kinase, delta) | - | Yes | - | - |
| 815 | CAMK2A | 5q33.1 | calcium/calmodulin-dependent protein kinase (CaM kinase) II alpha | - | - | Yes | - |
| 826 | CAPNS1 | 19q13.12 | calpain, small subunit 1 | Yes | Yes | - | - |
| 832 | CAPZB | 1p36.1 | capping protein (actin filament) muscle Z-line, beta | Yes | Yes | - | - |
| 836 | CASP3 | 4q34 | caspase 3, apoptosis-related cysteine peptidase | - | - | Yes | Yes |
| 885 | CCK | 3p22-p21.3 | cholecystokinin | Yes | Yes | - | - |
| 1020 | CDK5 | 7q36 | cyclin-dependent kinase 5 | Yes | - | - | - |
| 1026 | CDKN1A | 6p21.2 | cyclin-dependent kinase inhibitor 1A (p21, Cip1) | - | Yes | - | - |
| 1052 | CEBPD | 8p11.2-p11.1 | CCAAT/enhancer binding protein (C/EBP), delta | - | Yes | - | - |
| 1124 | CHN2 | 7p15.3 | chimerin (chimaerin) 2 | Yes | Yes | Yes | Yes |
| 1137 | CHRNA4 | 20q13.2-q13.3 | cholinergic receptor, nicotinic, alpha 4 | - | - | - | Yes |
| 1139 | CHRNA7 | 15q14 | cholinergic receptor, nicotinic, alpha 7 | - | Yes | - | - |
| 1152 | CKB | 14q32 | creatine kinase, brain | - | Yes | - | Yes |
| 1182 | CLCN3 | 4q33 | chloride channel 3 | - | Yes | - | - |
| 1209 | CLPTM1 | 19q13.2-q13.3 | cleft lip and palate associated transmembrane protein 1 | - | Yes | - | - |
| 1212 | CLTB | 4q2-q3\|5q35 | clathrin, light polypeptide (Lcb) | Yes | - | - | - |
| 1230 | CCR1 | 3p21 | chemokine (C-C motif) receptor 1 | - | Yes | - | - |
| 1234 | CCR5 | 3p21 | chemokine (C-C motif) receptor 5 | Yes | Yes | Yes | - |
| 1267 | CNP | 17q21 | 2',3'-cyclic nucleotide 3' phosphodiesterase | - | Yes | - | Yes |
| 1268 | CNR1 | 6q14-q15 | cannabinoid receptor 1 (brain) | Yes | Yes | Yes | Yes |
| 1278 | COL1A2 | 7q22.1 | collagen, type I, alpha 2 | - | Yes | - | - |
| 1345 | COX6C | 8q22-q23 | cytochrome c oxidase subunit VIc | Yes | Yes | - | - |
| 1347 | COX7A2 | 6q12 | cytochrome c oxidase subunit VIIa polypeptide 2 (liver) | Yes | Yes | Yes | - |
| 1363 | CPE | 4q32.3 | carboxypeptidase E | - | Yes | - | - |
| 1366 | CLDN7 | 17p13 | claudin 7 | - | Yes | - | - |
| 1381 | CRABP1 | 15q24 | cellular retinoic acid binding protein 1 | - | Yes | - | - |
| 1382 | CRABP2 | 1q21.3 | cellular retinoic acid binding protein 2 | - | Yes | - | - |
| 1385 | CREB1 | 2q34 | cAMP responsive element binding protein 1 | Yes | Yes | Yes | Yes |
| 1390 | CREM | 10p11.21 | cAMP responsive element modulator | - | Yes | - | - |
| 1409 | CRYAA | 21q22.3 | crystallin, alpha A | - | Yes | - | - |
| 1410 | CRYAB | 11q22.3-q23.1 | crystallin, alpha B | Yes | Yes | Yes | - |
| 1428 | CRYM | 16p13.11-p12.3 | crystallin, mu | - | Yes | - | Yes |
| 1490 | CTGF | 6q23.1 | connective tissue growth factor | - | Yes | - | - |
| 1499 | CTNNB1 | 3p21 | catenin (cadherin-associated protein), beta 1, 88kDa | Yes | Yes | - | - |
| 1501 | CTNND2 | 5p15.2 | catenin (cadherin-associated protein), delta 2 (neural plakophilin-related arm-repeat protein) | Yes | Yes | Yes | Yes |
| 1548 | CYP2A6 | 19q13.2 | cytochrome P450, family 2, subfamily A, polypeptide 6 | - | - | - | Yes |
| 1621 | DBH | 9q34 | dopamine beta-hydroxylase (dopamine beta-monooxygenase) | - | Yes | - | - |
| 1745 | DLX1 | 2q32 | distal-less homeobox 1 | - | Yes | - | - |
| 1813 | DRD2 | 11q23 | dopamine receptor D2 | Yes | Yes | Yes | Yes |
| 1814 | DRD3 | 3q13.3 | dopamine receptor D3 | Yes | - | - | - |
| 1815 | DRD4 | 11p15.5 | dopamine receptor D4 | Yes | Yes | Yes | Yes |
| 1848 | DUSP6 | 12q22-q23 | dual specificity phosphatase 6 | - | Yes | - | - |
| 1902 | EDG2 | 9q31.3 | endothelial differentiation, lysophosphatidic acid G-protein-coupled receptor, 2 | Yes | Yes | - | - |
| 1958 | EGR1 | 5q31.1 | early growth response 1 | Yes | Yes | - | - |
| 1959 | EGR2 | 10q21.1 | early growth response 2 (Krox-20 homolog, Drosophila) | Yes | - | Yes | - |
| 2012 | EMP1 | 12p12.3 | epithelial membrane protein 1 | - | Yes | - | - |
| 2023 | ENO1 | 1p36.3-p36.2 | enolase 1, (alpha) | Yes | Yes | - | - |
| 2026 | ENO2 | 12p13 | enolase 2 (gamma, neuronal) | Yes | Yes | Yes | - |
| 2027 | ENO3 | 17pter-p11 | enolase 3 (beta, muscle) | - | - | Yes | - |
| 2065 | ERBB3 | 12q13 | v-erb-b2 erythroblastic leukemia viral oncogene homolog 3 (avian) | - | Yes | - | - |
| 2119 | ETV5 | 3q28 | ets variant gene 5 (ets-related molecule) | Yes | Yes | Yes | Yes |
| 2166 | FAAH | 1p35-p34 | fatty acid amide hydrolase | Yes | Yes | Yes | Yes |
| 2173 | FABP7 | 6q22-q23 | fatty acid binding protein 7, brain | - | Yes | - | - |
| 2247 | FGF2 | 4q26-q27 | fibroblast growth factor 2 (basic) | Yes | Yes | - | - |
| 2289 | FKBP5 | 6p21.3-21.2 | FK506 binding protein 5 | - | Yes | - | - |
| 2335 | FN1 | 2q34 | fibronectin 1 | - | Yes | - | - |
| 2353 | FOS | 14q24.3 | v-fos FBJ murine osteosarcoma viral oncogene homolog | Yes | Yes | Yes | Yes |
| 2354 | FOSB | 19q13.32 | FBJ murine osteosarcoma viral oncogene homolog B | Yes | Yes | Yes | Yes |
| 2359 | FPRL2 | 19q13.3-q13.4 | formyl peptide receptor-like 2 | - | Yes | - | - |
| 2548 | GAA | 17q25.2-q25.3 | glucosidase, alpha; acid (Pompe disease, glycogen storage disease type II) | Yes | Yes | - | - |
| 2550 | GABBR1 | 6p21.31 | gamma-aminobutyric acid (GABA) B receptor, 1 | Yes | Yes | - | - |
| 2560 | GABRB1 | 4p12 | gamma-aminobutyric acid (GABA) A receptor, beta 1 | Yes | Yes | - | - |
| 2565 | GABRG1 | 4p12 | gamma-aminobutyric acid (GABA) A receptor, gamma 1 | Yes | Yes | Yes | Yes |
| 2566 | GABRG2 | 5q31.1-q33.1 | gamma-aminobutyric acid (GABA) A receptor, gamma 2 | - | Yes | - | - |
| 2571 | GAD1 | 2q31 | glutamate decarboxylase 1 (brain, 67kDa) | Yes | - | - | - |
| 2597 | GAPDH | 12p13 | glyceraldehyde-3-phosphate dehydrogenase | - | Yes | Yes | - |
| 2617 | GARS | 7p15 | glycyl-tRNA synthetase | - | Yes | - | - |
| 2657 | GDF1 | 19p12 | growth differentiation factor 1 | - | Yes | - | - |
| 2668 | GDNF | 5p13.1-p12 | glial cell derived neurotrophic factor | Yes | - | Yes | - |
| 2670 | GFAP | 17q21 | glial fibrillary acidic protein | - | Yes | - | - |
| 2729 | GCLC | 6p12 | glutamate-cysteine ligase, catalytic subunit | Yes | Yes | Yes | - |
| 2746 | GLUD1 | 10q23.3 | glutamate dehydrogenase 1 | - | Yes | Yes | - |
| 2767 | GNA11 | 19p13.3 | guanine nucleotide binding protein (G protein), alpha 11 (Gq class) | Yes | - | - | - |
| 2770 | GNAI1 | 7q21 | guanine nucleotide binding protein (G protein), alpha inhibiting activity polypeptide 1 | - | - | Yes | - |
| 2775 | GNAO1 | 16q13 | guanine nucleotide binding protein (G protein), alpha activating activity polypeptide O | Yes | Yes | Yes | - |
| 2776 | GNAQ | 9q21 | guanine nucleotide binding protein (G protein), q polypeptide | Yes | - | - | - |
| 2782 | GNB1 | 1p36.33 | guanine nucleotide binding protein (G protein), beta polypeptide 1 | Yes | - | Yes | - |
| 2824 | GPM6B | Xp22.2 | glycoprotein M6B | Yes | Yes | - | - |
| 2885 | GRB2 | 17q24-q25 | growth factor receptor-bound protein 2 | Yes | Yes | - | - |
| 2891 | GRIA2 | 4q32-q33 | glutamate receptor, ionotropic, AMPA 2 | Yes | Yes | - | - |
| 2897 | GRIK1 | 21q22.11 | glutamate receptor, ionotropic, kainate 1 | Yes | - | - | - |
| 2901 | GRIK5 | 19q13.2 | glutamate receptor, ionotropic, kainate 5 | Yes | - | - | - |
| 2902 | GRIN1 | 9q34.3 | glutamate receptor, ionotropic, N-methyl D-aspartate 1 | Yes | - | - | - |
| 2905 | GRIN2C | 17q25 | glutamate receptor, ionotropic, N-methyl D-aspartate 2C | Yes | Yes | Yes | - |
| 2907 | GRINA | 8q24.3 | glutamate receptor, ionotropic, N-methyl D-asparate-associated protein 1 (glutamate binding) | Yes | Yes | - | - |
| 2908 | NR3C1 | 5q31.3 | nuclear receptor subfamily 3, group C, member 1 (glucocorticoid receptor) | Yes | Yes | Yes | Yes |
| 2915 | GRM5 | 11q14.2-q14.3 | glutamate receptor, metabotropic 5 | Yes | - | - | - |
| 2923 | PDIA3 | 15q15 | protein disulfide isomerase family A, member 3 | Yes | Yes | - | - |
| 2950 | GSTP1 | 11q13 | glutathione S-transferase pi | - | Yes | - | - |
| 3091 | HIF1A | 14q21-q24 | hypoxia-inducible factor 1, alpha subunit (basic helix-loop-helix transcription factor) | Yes | Yes | Yes | Yes |
| 3107 | HLA-C | 6p21.3 | major histocompatibility complex, class I, C | Yes | - | - | - |
| 3122 | HLA-DRA | 6p21.3 | major histocompatibility complex, class II, DR alpha | - | Yes | - | - |
| 3127 | HLA-DRB5 | 6p21.3 | major histocompatibility complex, class II, DR beta 5 | - | - | Yes | - |
| 3156 | HMGCR | 5q13.3-q14 | 3-hydroxy-3-methylglutaryl-Coenzyme A reductase | - | Yes | Yes | - |
| 3162 | HMOX1 | 22q12\|22q13.1 | heme oxygenase (decycling) 1 | - | Yes | - | - |
| 3164 | NR4A1 | 12q13 | nuclear receptor subfamily 4, group A, member 1 | Yes | Yes | Yes | Yes |
| 3303 | HSPA1A | 6p21.3 | heat shock 70kDa protein 1A | - | - | Yes | - |
| 3304 | HSPA1B | 6p21.3 | heat shock 70kDa protein 1B | - | - | Yes | - |
| 3309 | HSPA5 | 9q33-q34.1 | heat shock 70kDa protein 5 (glucose-regulated protein, 78kDa) | Yes | - | Yes | - |
| 3312 | HSPA8 | 11q24.1 | heat shock 70kDa protein 8 | - | Yes | - | - |
| 3315 | HSPB1 | 7q11.23 | heat shock 27kDa protein 1 | - | - | Yes | - |
| 3316 | HSPB2 | 11q22-q23 | heat shock 27kDa protein 2 | Yes | Yes | - | - |
| 3337 | DNAJB1 | 19p13.2 | DnaJ (Hsp40) homolog, subfamily B, member 1 | - | - | Yes | - |
| 3351 | HTR1B | 6q13 | 5-hydroxytryptamine (serotonin) receptor 1B | Yes | Yes | Yes | - |
| 3356 | HTR2A | 13q14-q21 | 5-hydroxytryptamine (serotonin) receptor 2A | - | - | - | Yes |
| 3480 | IGF1R | 15q26.3 | insulin-like growth factor 1 receptor | - | Yes | - | - |
| 3481 | IGF2 | 11p15.5 | insulin-like growth factor 2 (somatomedin A) | Yes | Yes | - | Yes |
| 3485 | IGFBP2 | 2q33-q34 | insulin-like growth factor binding protein 2, 36kDa | Yes | Yes | - | - |
| 3568 | IL5RA | 3p26-p24 | interleukin 5 receptor, alpha | - | Yes | - | - |
| 3587 | IL10RA | 11q23 | interleukin 10 receptor, alpha | Yes | Yes | - | - |
| 3604 | TNFRSF9 | 1p36 | tumor necrosis factor receptor superfamily, member 9 | - | Yes | - | - |
| 3707 | ITPKB | 1q42.13 | inositol 1,4,5-trisphosphate 3-kinase B | Yes | Yes | - | - |
| 3725 | JUN | 1p32-p31 | v-jun sarcoma virus 17 oncogene homolog (avian) | Yes | Yes | - | - |
| 3726 | JUNB | 19p13.2 | jun B proto-oncogene | Yes | - | - | - |
| 3751 | KCND2 | 7q31 | potassium voltage-gated channel, Shal-related subfamily, member 2 | Yes | Yes | - | - |
| 3766 | KCNJ10 | 1q22-q23 | potassium inwardly-rectifying channel, subfamily J, member 10 | Yes | Yes | - | - |
| 3778 | KCNMA1 | 10q22.3 | potassium large conductance calcium-activated channel, subfamily M, alpha member 1 | - | Yes | - | - |
| 3916 | LAMP1 | 13q34 | lysosomal-associated membrane protein 1 | Yes | Yes | Yes | Yes |
| 3921 | RPSA | 3p22.2 | ribosomal protein SA | Yes | Yes | - | - |
| 3987 | LIMS1 | 2q12.3-q13 | LIM and senescent cell antigen-like domains 1 | - | Yes | - | - |
| 4034 | LRCH4 | 7q22 | leucine-rich repeats and calponin homology (CH) domain containing 4 | - | Yes | - | - |
| 4057 | LTF | 3q21-q23 | lactotransferrin | - | Yes | - | - |
| 4069 | LYZ | 12q15 | lysozyme (renal amyloidosis) | - | Yes | Yes | - |
| 4070 | TACSTD2 | 1p32-p31 | tumor-associated calcium signal transducer 2 | - | Yes | - | - |
| 4099 | MAG | 19q13.1 | myelin associated glycoprotein | - | Yes | - | - |
| 4118 | MAL | 2cen-q13 | mal, T-cell differentiation protein | - | Yes | - | - |
| 4129 | MAOB | Xp11.23 | monoamine oxidase B | - | - | - | Yes |
| 4131 | MAP1B | 5q13 | microtubule-associated protein 1B | Yes | - | Yes | - |
| 4137 | MAPT | 17q21.1 | microtubule-associated protein tau | Yes | Yes | - | - |
| 4155 | MBP | 18q23 | myelin basic protein | Yes | Yes | Yes | Yes |
| 4191 | MDH2 | 7p12.3-q11.2 | malate dehydrogenase 2, NAD (mitochondrial) | - | - | Yes | - |
| 4208 | MEF2C | 5q14 | MADS box transcription enhancer factor 2, polypeptide C (myocyte enhancer factor 2C) | - | Yes | - | - |
| 4299 | AFF1 | 4q21 | AF4/FMR2 family, member 1 | - | Yes | - | - |
| 4336 | MOBP | 3p22.1 | myelin-associated oligodendrocyte basic protein | Yes | Yes | - | - |
| 4340 | MOG | 6p22.1 | myelin oligodendrocyte glycoprotein | - | Yes | - | - |
| 4353 | MPO | 17q23.1 | myeloperoxidase | - | Yes | - | Yes |
| 4359 | MPZ | 1q22 | myelin protein zero (Charcot-Marie-Tooth neuropathy 1B) | - | Yes | - | - |
| 4485 | MST1 | 3p21 | macrophage stimulating 1 (hepatocyte growth factor-like) | - | Yes | - | - |
| 4501 | MT1X | 16q13 | metallothionein 1X | - | Yes | Yes | - |
| 4502 | MT2A | 16q13 | metallothionein 2A | - | Yes | - | - |
| 4512 | COX1 | - | cytochrome c oxidase subunit I | Yes | - | - | - |
| 4719 | NDUFS1 | 2q33-q34 | NADH dehydrogenase (ubiquinone) Fe-S protein 1, 75kDa (NADH-coenzyme Q reductase) | Yes | Yes | - | - |
| 4729 | NDUFV2 | 18p11.31-p11.2 | NADH dehydrogenase (ubiquinone) flavoprotein 2, 24kDa | Yes | - | Yes | - |
| 4741 | NEF3 | 8p21 | neurofilament 3 (150kDa medium) | Yes | Yes | - | - |
| 4744 | NEFH | 22q12.2 | neurofilament, heavy polypeptide 200kDa | Yes | Yes | - | - |
| 4747 | NEFL | 8p21 | neurofilament, light polypeptide 68kDa | Yes | - | Yes | - |
| 4753 | NELL2 | 12q13.11-q13.12 | NEL-like 2 (chicken) | - | Yes | - | - |
| 4763 | NF1 | 17q11.2 | neurofibromin 1 (neurofibromatosis, von Recklinghausen disease, Watson disease) | Yes | - | Yes | - |
| 4774 | NFIA | 1p31.3-p31.2 | nuclear factor I/A | Yes | Yes | Yes | - |
| 4781 | NFIB | 9p24.1 | nuclear factor I/B | Yes | Yes | Yes | Yes |
| 4792 | NFKBIA | 14q13 | nuclear factor of kappa light polypeptide gene enhancer in B-cells inhibitor, alpha | - | Yes | Yes | - |
| 4821 | NKX2-2 | 20pter-q11.23 | NK2 transcription factor related, locus 2 (Drosophila) | - | Yes | - | - |
| 4852 | NPY | 7p15.1 | neuropeptide Y | Yes | Yes | - | - |
| 4884 | NPTX1 | 17q25.1-q25.2 | neuronal pentraxin I | Yes | Yes | - | - |
| 4900 | NRGN | 11q24 | neurogranin (protein kinase C substrate, RC3) | Yes | Yes | - | - |
| 4905 | NSF | 17q21 | N-ethylmaleimide-sensitive factor | Yes | Yes | Yes | - |
| 4915 | NTRK2 | 9q22.1 | neurotrophic tyrosine kinase, receptor, type 2 | Yes | Yes | Yes | - |
| 4985 | OPRD1 | 1p36.1-p34.3 | opioid receptor, delta 1 | - | - | Yes | - |
| 4986 | OPRK1 | 8q11.2 | opioid receptor, kappa 1 | Yes | - | Yes | - |
| 4988 | OPRM1 | 6q24-q25 | opioid receptor, mu 1 | - | - | Yes | - |
| 5066 | PAM | 5q14-q21 | peptidylglycine alpha-amidating monooxygenase | - | Yes | - | - |
| 5091 | PC | 11q13.4-q13.5 | pyruvate carboxylase | - | Yes | - | - |
| 5121 | PCP4 | 21q22.2 | Purkinje cell protein 4 | Yes | Yes | Yes | Yes |
| 5126 | PCSK2 | 20p11.2 | proprotein convertase subtilisin/kexin type 2 | Yes | Yes | Yes | Yes |
| 5166 | PDK4 | 7q21.3 | pyruvate dehydrogenase kinase, isozyme 4 | - | Yes | - | - |
| 5168 | ENPP2 | 8q24.1 | ectonucleotide pyrophosphatase/phosphodiesterase 2 (autotaxin) | - | Yes | - | - |
| 5173 | PDYN | 20pter-p12 | prodynorphin | Yes | Yes | Yes | - |
| 5179 | PENK | 8q23-q24 | proenkephalin | Yes | Yes | Yes | Yes |
| 5187 | PER1 | 17p13.1-17p12 | period homolog 1 (Drosophila) | Yes | - | - | - |
| 5315 | PKM2 | 15q22 | pyruvate kinase, muscle | Yes | - | Yes | - |
| 5319 | PLA2G1B | 12q23-q24.1 | phospholipase A2, group IB (pancreas) | - | Yes | - | - |
| 5354 | PLP1 | Xq22 | proteolipid protein 1 (Pelizaeus-Merzbacher disease, spastic paraplegia 2, uncomplicated) | Yes | Yes | - | - |
| 5376 | PMP22 | 17p12-p11.2 | peripheral myelin protein 22 | - | Yes | - | - |
| 5414 | 4-Sep | 17q22-q23 | septin 4 | Yes | - | - | Yes |
| 5495 | PPM1B | 2p21 | protein phosphatase 1B (formerly 2C), magnesium-dependent, beta isoform | - | Yes | - | - |
| 5504 | PPP1R2 | 3q29 | protein phosphatase 1, regulatory (inhibitor) subunit 2 | - | Yes | - | - |
| 5516 | PPP2CB | 8p12 | protein phosphatase 2 (formerly 2A), catalytic subunit, beta isoform | Yes | Yes | - | - |
| 5527 | PPP2R5C | 14q32 | protein phosphatase 2, regulatory subunit B (B56), gamma isoform | - | Yes | - | - |
| 5573 | PRKAR1A | 17q23-q24 | protein kinase, cAMP-dependent, regulatory, type I, alpha (tissue specific extinguisher 1) | - | Yes | - | - |
| 5575 | PRKAR1B | 7p22 | protein kinase, cAMP-dependent, regulatory, type I, beta | - | Yes | - | - |
| 5578 | PRKCA | 17q22-q23.2 | protein kinase C, alpha | Yes | - | - | - |
| 5581 | PRKCE | 2p21 | protein kinase C, epsilon | Yes | - | - | - |
| 5582 | PRKCG | 19q13.4 | protein kinase C, gamma | Yes | - | Yes | - |
| 5590 | PRKCZ | 1p36.33-p36.2 | protein kinase C, zeta | Yes | Yes | - | - |
| 5594 | MAPK1 | 22q11.2\|22q11.21 | mitogen-activated protein kinase 1 | Yes | Yes | - | - |
| 5595 | MAPK3 | 16p11.2 | mitogen-activated protein kinase 3 | Yes | - | - | - |
| 5601 | MAPK9 | 5q35 | mitogen-activated protein kinase 9 | - | Yes | Yes | - |
| 5602 | MAPK10 | 4q22.1-q23 | mitogen-activated protein kinase 10 | Yes | Yes | Yes | Yes |
| 5604 | MAP2K1 | 15q22.1-q22.33 | mitogen-activated protein kinase kinase 1 | Yes | Yes | - | - |
| 5654 | HTRA1 | 10q26.3 | HtrA serine peptidase 1 | - | Yes | - | - |
| 5692 | PSMB4 | 1q21 | proteasome (prosome, macropain) subunit, beta type, 4 | - | - | Yes | - |
| 5747 | PTK2 | 8q24-qter | PTK2 protein tyrosine kinase 2 | Yes | Yes | - | - |
| 5813 | PURA | 5q31 | purine-rich element binding protein A | - | Yes | - | - |
| 5860 | QDPR | 4p15.31 | quinoid dihydropteridine reductase | Yes | Yes | Yes | Yes |
| 5870 | RAB6A | 11q13.3 | RAB6A, member RAS oncogene family | - | Yes | - | Yes |
| 5887 | RAD23B | 9q31.2 | RAD23 homolog B (S. cerevisiae) | - | Yes | - | - |
| 5901 | RAN | 12q24.3 | RAN, member RAS oncogene family | - | Yes | - | - |
| 5925 | RB1 | 13q14.2 | retinoblastoma 1 (including osteosarcoma) | - | Yes | - | - |
| 5977 | DPF2 | 11q13 | D4, zinc and double PHD fingers family 2 | - | Yes | - | - |
| 5999 | RGS4 | 1q23.3 | regulator of G-protein signalling 4 | Yes | Yes | Yes | Yes |
| 6122 | RPL3 | 22q13 | ribosomal protein L3 | - | Yes | - | - |
| 6147 | RPL23A | 17q11 | ribosomal protein L23a | Yes | - | - | - |
| 6156 | RPL30 | 8q22 | ribosomal protein L30 | - | Yes | - | - |
| 6199 | RPS6KB2 | 11q13.1 | ribosomal protein S6 kinase, 70kDa, polypeptide 2 | - | Yes | - | - |
| 6252 | RTN1 | 14q23.1 | reticulon 1 | - | Yes | - | - |
| 6310 | ATXN1 | 6p23 | ataxin 1 | - | Yes | - | - |
| 6327 | SCN2B | 11q23 | sodium channel, voltage-gated, type II, beta | Yes | Yes | - | - |
| 6376 | CX3CL1 | 16q13 | chemokine (C-X3-C motif) ligand 1 | Yes | Yes | - | - |
| 6383 | SDC2 | 8q22-q23 | syndecan 2 (heparan sulfate proteoglycan 1, cell surface-associated, fibroglycan) | - | Yes | - | - |
| 6387 | CXCL12 | 10q11.1 | chemokine (C-X-C motif) ligand 12 (stromal cell-derived factor 1) | - | Yes | - | - |
| 6421 | SFPQ | 1p34.3 | splicing factor proline/glutamine-rich (polypyrimidine tract binding protein associated) | Yes | Yes | Yes | Yes |
| 6431 | SFRS6 | 20q12-q13.1 | splicing factor, arginine/serine-rich 6 | - | Yes | - | - |
| 6432 | SFRS7 | 2p22.1 | splicing factor, arginine/serine-rich 7, 35kDa | - | Yes | - | - |
| 6446 | SGK | 6q23 | serum/glucocorticoid regulated kinase | - | Yes | Yes | - |
| 6513 | SLC2A1 | 1p35-p31.3 | solute carrier family 2 (facilitated glucose transporter), member 1 | - | Yes | - | - |
| 6520 | SLC3A2 | 11q13 | solute carrier family 3 (activators of dibasic and neutral amino acid transport), member 2 | - | Yes | - | - |
| 6529 | SLC6A1 | 3p25-p24 | solute carrier family 6 (neurotransmitter transporter, GABA), member 1 | - | Yes | Yes | - |
| 6530 | SLC6A2 | 16q12.2 | solute carrier family 6 (neurotransmitter transporter, noradrenalin), member 2 | - | Yes | - | - |
| 6531 | SLC6A3 | 5p15.3 | solute carrier family 6 (neurotransmitter transporter, dopamine), member 3 | - | - | - | Yes |
| 6532 | SLC6A4 | 17q11.1-q12 | solute carrier family 6 (neurotransmitter transporter, serotonin), member 4 | Yes | Yes | Yes | Yes |
| 6602 | SMARCD1 | 12q13-q14 | SWI/SNF related, matrix associated, actin dependent regulator of chromatin, subfamily d, member 1 | - | Yes | - | - |
| 6606 | SMN1 | 5q13 | survival of motor neuron 1, telomeric | Yes | Yes | Yes | Yes |
| 6610 | SMPD2 | 6q21 | sphingomyelin phosphodiesterase 2, neutral membrane (neutral sphingomyelinase) | - | Yes | - | - |
| 6620 | SNCB | 5q35 | synuclein, beta | - | Yes | - | - |
| 6622 | SNCA | 4q21 | synuclein, alpha (non A4 component of amyloid precursor) | - | Yes | - | - |
| 6624 | FSCN1 | 7p22 | fascin homolog 1, actin-bundling protein (Strongylocentrotus purpuratus) | - | Yes | - | - |
| 6648 | SOD2 | 6q25.3 | superoxide dismutase 2, mitochondrial | - | Yes | - | - |
| 6657 | SOX2 | 3q26.3-q27 | SRY (sex determining region Y)-box 2 | - | Yes | - | - |
| 6662 | SOX9 | 17q24.3-q25.1 | SRY (sex determining region Y)-box 9 (campomelic dysplasia, autosomal sex-reversal) | - | Yes | - | - |
| 6663 | SOX10 | 22q13.1 | SRY (sex determining region Y)-box 10 | - | Yes | - | - |
| 6678 | SPARC | 5q31.3-q32 | secreted protein, acidic, cysteine-rich (osteonectin) | Yes | Yes | - | - |
| 6712 | SPTBN2 | 11q13 | spectrin, beta, non-erythrocytic 2 | - | Yes | - | - |
| 6804 | STX1A | 7q11.23 | syntaxin 1A (brain) | - | Yes | - | - |
| 6813 | STXBP2 | 19p13.3-p13.2 | syntaxin binding protein 2 | - | Yes | - | - |
| 6853 | SYN1 | Xp11.23 | synapsin I | - | - | Yes | - |
| 6854 | SYN2 | 3p25 | synapsin II | - | Yes | - | - |
| 6863 | TAC1 | 7q21-q22 | tachykinin, precursor 1 (substance K, substance P, neurokinin 1, neurokinin 2, neuromedin L, neurokinin alpha, neuropeptide K, neuropeptide gamma) | Yes | Yes | Yes | - |
| 6869 | TACR1 | 2p13.1 | tachykinin receptor 1 | - | - | Yes | - |
| 7001 | PRDX2 | 19p13.2 | peroxiredoxin 2 | - | Yes | - | - |
| 7018 | TF | 3q22.1 | transferrin | - | Yes | - | - |
| 7021 | TFAP2B | 6p21-p12 | transcription factor AP-2 beta (activating enhancer binding protein 2 beta) | - | Yes | - | - |
| 7025 | NR2F1 | 5q14 | nuclear receptor subfamily 2, group F, member 1 | - | Yes | - | - |
| 7037 | TFRC | 3q29 | transferrin receptor (p90, CD71) | Yes | Yes | - | - |
| 7054 | TH | 11p15.5 | tyrosine hydroxylase | Yes | Yes | - | Yes |
| 7068 | THRB | 3p24.2 | thyroid hormone receptor, beta (erythroblastic leukemia viral (v-erb-a) oncogene homolog 2, avian) | - | Yes | - | - |
| 7070 | THY1 | 11q22.3-q23 | Thy-1 cell surface antigen | Yes | Yes | - | - |
| 7071 | KLF10 | 8q22.2 | Kruppel-like factor 10 | - | Yes | Yes | - |
| 7076 | TIMP1 | Xp11.3-p11.23 | TIMP metallopeptidase inhibitor 1 | - | Yes | - | - |
| 7080 | TITF1 | 14q13 | thyroid transcription factor 1 | - | Yes | - | - |
| 7086 | TKT | 3p14.3 | transketolase (Wernicke-Korsakoff syndrome) | Yes | Yes | Yes | Yes |
| 7122 | CLDN5 | 22q11.21 | claudin 5 (transmembrane protein deleted in velocardiofacial syndrome) | - | Yes | - | - |
| 7124 | TNF | 6p21.3 | tumor necrosis factor (TNF superfamily, member 2) | - | - | - | Yes |
| 7167 | TPI1 | 12p13 | triosephosphate isomerase 1 | Yes | - | Yes | - |
| 7277 | TUBA1 | 2q35 | tubulin, alpha 1 (testis specific) | Yes | Yes | - | - |
| 7287 | TULP1 | 6p21.3 | tubby like protein 1 | - | Yes | - | - |
| 7336 | UBE2V2 | 8q11.21 | ubiquitin-conjugating enzyme E2 variant 2 | Yes | Yes | - | - |
| 7345 | UCHL1 | 4p14 | ubiquitin carboxyl-terminal esterase L1 (ubiquitin thiolesterase) | Yes | Yes | Yes | - |
| 7384 | UQCRC1 | 3p21.3 | ubiquinol-cytochrome c reductase core protein I | Yes | Yes | - | - |
| 7385 | UQCRC2 | 16p12 | ubiquinol-cytochrome c reductase core protein II | - | Yes | - | - |
| 7416 | VDAC1 | 5q31 | voltage-dependent anion channel 1 | Yes | Yes | - | - |
| 7447 | VSNL1 | 2p24.3 | visinin-like 1 | - | Yes | - | - |
| 7514 | XPO1 | 2p16 | exportin 1 (CRM1 homolog, yeast) | - | Yes | - | - |
| 7529 | YWHAB | 20q13.1 | tyrosine 3-monooxygenase/tryptophan 5-monooxygenase activation protein, beta polypeptide | - | Yes | - | Yes |
| 7532 | YWHAG | 7q11.23 | tyrosine 3-monooxygenase/tryptophan 5-monooxygenase activation protein, gamma polypeptide | - | Yes | Yes | - |
| 7533 | YWHAH | 22q12.3 | tyrosine 3-monooxygenase/tryptophan 5-monooxygenase activation protein, eta polypeptide | Yes | Yes | - | - |
| 7704 | ZBTB16 | 11q23.1 | zinc finger and BTB domain containing 16 | - | Yes | - | - |
| 7846 | TUBA3 | 12q12-12q14.3 | tubulin, alpha 3 | Yes | Yes | - | - |
| 7852 | CXCR4 | 2q21 | chemokine (C-X-C motif) receptor 4 | Yes | Yes | - | - |
| 7857 | SCG2 | 2q35-q36 | secretogranin II (chromogranin C) | - | Yes | - | - |
| 8013 | NR4A3 | 9q22 | nuclear receptor subfamily 4, group A, member 3 | Yes | - | Yes | - |
| 8100 | IFT88 | 13q12.1 | intraflagellar transport 88 homolog (Chlamydomonas) | - | Yes | - | - |
| 8288 | EPX | 17q23.1 | eosinophil peroxidase | - | Yes | - | Yes |
| 8326 | FZD9 | 7q11.23 | frizzled homolog 9 (Drosophila) | - | Yes | - | - |
| 8439 | NSMAF | 8q12-q13 | neutral sphingomyelinase (N-SMase) activation associated factor | - | Yes | - | - |
| 8775 | NAPA | 19q13.32 | N-ethylmaleimide-sensitive factor attachment protein, alpha | - | - | Yes | - |
| 8777 | MPDZ | 9p24-p22 | multiple PDZ domain protein | - | Yes | - | - |
| 8787 | RGS9 | 17q23-q24 | regulator of G-protein signalling 9 | - | Yes | - | - |
| 8829 | NRP1 | 10p12 | neuropilin 1 | - | Yes | - | - |
| 8864 | PER2 | 2q37.3 | period homolog 2 (Drosophila) | Yes | - | Yes | - |
| 8993 | PGLYRP1 | 19q13.2-q13.3 | peptidoglycan recognition protein 1 | - | Yes | - | - |
| 8997 | KALRN | 3q21.1-q21.2 | kalirin, RhoGEF kinase | - | Yes | - | - |
| 9040 | UBE2M | 19q13.43 | ubiquitin-conjugating enzyme E2M (UBC12 homolog, yeast) | Yes | Yes | Yes | Yes |
| 9146 | HGS | 17q25 | hepatocyte growth factor-regulated tyrosine kinase substrate | Yes | Yes | - | - |
| 9230 | RAB11B | 19p13.2 | RAB11B, member RAS oncogene family | - | Yes | - | - |
| 9241 | NOG | 17q21-q22 | noggin | - | - | Yes | - |
| 9252 | RPS6KA5 | 14q31-q32.1 | ribosomal protein S6 kinase, 90kDa, polypeptide 5 | Yes | Yes | - | - |
| 9322 | TRIP10 | 19p13.3 | thyroid hormone receptor interactor 10 | - | Yes | - | - |
| 9341 | VAMP3 | 1p36.23 | vesicle-associated membrane protein 3 (cellubrevin) | - | Yes | - | - |
| 9452 | ITM2A | Xq13.3-Xq21.2 | integral membrane protein 2A | - | Yes | - | - |
| 9455 | HOMER2 | 15q24.3 | homer homolog 2 (Drosophila) | Yes | - | - | - |
| 9527 | GOSR1 | 17q11 | golgi SNAP receptor complex member 1 | - | Yes | - | - |
| 9568 | GABBR2 | 9q22.1-q22.3 | gamma-aminobutyric acid (GABA) B receptor, 2 | - | - | - | Yes |
| 9575 | CLOCK | 4q12 | clock homolog (mouse) | Yes | - | - | - |
| 9588 | PRDX6 | 1q25.1 | peroxiredoxin 6 | - | Yes | Yes | - |
| 9607 | CART | 5q13.2 | cocaine- and amphetamine-regulated transcript | Yes | - | - | - |
| 9615 | GDA | 9q21.13 | guanine deaminase | - | - | Yes | - |
| 9868 | TOMM70A | 3q12.2 | translocase of outer mitochondrial membrane 70 homolog A (S. cerevisiae) | - | Yes | - | - |
| 10076 | PTPRU | 1p35.3-p35.1 | protein tyrosine phosphatase, receptor type, U | - | Yes | - | - |
| 10276 | NET1 | 10p15 | neuroepithelial cell transforming gene 1 | - | Yes | - | - |
| 10379 | ISGF3G | 14q11.2 | interferon-stimulated transcription factor 3, gamma 48kDa | Yes | - | - | - |
| 10399 | GNB2L1 | 5q35.3 | guanine nucleotide binding protein (G protein), beta polypeptide 2-like 1 | Yes | Yes | Yes | Yes |
| 10439 | OLFM1 | 9q34.3 | olfactomedin 1 | Yes | Yes | Yes | Yes |
| 10486 | CAP2 | 6p22.3 | CAP, adenylate cyclase-associated protein, 2 (yeast) | - | Yes | - | - |
| 10628 | TXNIP | 1q21.1 | thioredoxin interacting protein | - | Yes | - | - |
| 10681 | GNB5 | 15q21.2 | guanine nucleotide binding protein (G protein), beta 5 | Yes | Yes | Yes | - |
| 10772 | FUSIP1 | 1p36.11 | FUS interacting protein (serine/arginine-rich) 1 | - | Yes | - | - |
| 10844 | TUBGCP2 | 10q26.3 | tubulin, gamma complex associated protein 2 | - | Yes | - | - |
| 10912 | GADD45G | 9q22.1-q22.2 | growth arrest and DNA-damage-inducible, gamma | - | Yes | - | Yes |
| 10924 | SMPDL3A | 6q22.31 | sphingomyelin phosphodiesterase, acid-like 3A | - | Yes | - | - |
| 10950 | BTG3 | 21q21.1-q21.2 | BTG family, member 3 | - | Yes | - | - |
| 10972 | TMED10 | 14q24.3 | transmembrane emp24-like trafficking protein 10 (yeast) | - | Yes | - | - |
| 11031 | RAB31 | 18p11.3 | RAB31, member RAS oncogene family | - | Yes | - | - |
| 11075 | STMN2 | 8q21.13 | stathmin-like 2 | - | Yes | - | - |
| 11163 | NUDT4 | 12q21 | nudix (nucleoside diphosphate linked moiety X)-type motif 4 | - | Yes | - | - |
| 11170 | FAM107A | 3p21.1 | family with sequence similarity 107, member A | - | Yes | - | - |
| 22924 | MAPRE3 | 2p23.3-p23.1 | microtubule-associated protein, RP/EB family, member 3 | - | Yes | - | - |
| 23208 | SYT11 | 1q21.2 | synaptotagmin XI | Yes | Yes | - | - |
| 23236 | PLCB1 | 20p12 | phospholipase C, beta 1 (phosphoinositide-specific) | - | Yes | - | Yes |
| 23237 | ARC | 8q24.3 | activity-regulated cytoskeleton-associated protein | Yes | - | Yes | Yes |
| 23336 | DMN | 15q26.3 | desmuslin | - | Yes | - | - |
| 23451 | SF3B1 | 2q33.1 | splicing factor 3b, subunit 1, 155kDa | - | Yes | - | - |
| 23467 | NPTXR | 22q13.1 | neuronal pentraxin receptor | Yes | Yes | - | - |
| 23678 | SGK3 | 8q12.3-8q13.1 | serum/glucocorticoid regulated kinase family, member 3 | - | Yes | - | - |
| 25932 | CLIC4 | 1p36.11 | chloride intracellular channel 4 | - | Yes | - | - |
| 25946 | ZNF385 | 12q13.13 | zinc finger protein 385 | - | Yes | - | - |
| 27075 | TSPAN13 | 7p21.1 | tetraspanin 13 | Yes | Yes | Yes | Yes |
| 27086 | FOXP1 | 3p14.1 | forkhead box P1 | - | Yes | - | - |
| 27244 | SESN1 | 6q21 | sestrin 1 | - | Yes | - | - |
| 28951 | TRIB2 | 2p25.1-p24.3 | tribbles homolog 2 (Drosophila) | - | Yes | - | - |
| 28999 | KLF15 | 3q13-q21 | Kruppel-like factor 15 | - | Yes | - | - |
| 29114 | TAGLN3 | 3q13.2 | transgelin 3 | Yes | Yes | - | - |
| 29760 | BLNK | 10q23.2-q23.33 | B-cell linker | - | Yes | - | - |
| 29767 | TMOD2 | 15q21.1-q21.2 | tropomodulin 2 (neuronal) | Yes | Yes | Yes | - |
| 29896 | TRA2A | 7p15.3 | transformer-2 alpha | - | Yes | - | - |
| 29970 | SCHIP1 | 3q25.33 | schwannomin interacting protein 1 | - | Yes | - | - |
| 54206 | ERRFI1 | 1p36.12-36.33 | ERBB receptor feedback inhibitor 1 | - | Yes | - | - |
| 54541 | DDIT4 | 10pter-q26.12 | DNA-damage-inducible transcript 4 | - | Yes | - | - |
| 54704 | PPM2C | 8q22.1 | protein phosphatase 2C, magnesium-dependent, catalytic subunit | Yes | Yes | Yes | Yes |
| 54838 | C10orf26 | 10q24.32 | chromosome 10 open reading frame 26 | - | Yes | - | - |
| 54941 | RNF125 | 18q12.1 | ring finger protein 125 | - | Yes | - | - |
| 55319 | FLJ11184 | 4q32.3 | hypothetical protein FLJ11184 | - | Yes | - | - |
| 55885 | LMO3 | 12p12.3 | LIM domain only 3 (rhombotin-like 2) | Yes | Yes | Yes | - |
| 56253 | CRTAM | 11q22-q23 | class-I MHC-restricted T cell associated molecule | - | Yes | - | - |
| 58476 | TP53INP2 | 20q11.22 | tumor protein p53 inducible nuclear protein 2 | Yes | Yes | - | - |
| 58480 | RHOU | 1q42.11-q42.3 | ras homolog gene family, member U | - | Yes | - | - |
| 79830 | ZMYM1 | 1p34.3 | zinc finger, MYM-type 1 | Yes | Yes | Yes | Yes |
| 80176 | SPSB1 | 1p36.22 | splA/ryanodine receptor domain and SOCS box containing 1 | - | Yes | - | - |
| 94241 | TP53INP1 | 8q22 | tumor protein p53 inducible nuclear protein 1 | - | Yes | - | - |
| 112939 | BTBD14B | 19p13.13 | BTB (POZ) domain containing 14B | Yes | - | - | - |
| 113791 | MGC17330 | 22q12.2 | HGFL gene | - | Yes | - | - |
| 114823 | LENG8 | 19q13.42 | leukocyte receptor cluster (LRC) member 8 | - | Yes | - | - |
| 114876 | OSBPL1A | 18q11.1 | oxysterol binding protein-like 1A | Yes | Yes | Yes | Yes |
| 161060 | 窗体顶部  LOC161060  窗体底部 | 13 | similar to cytochrome c oxidase subunit III | - | Yes | - | - |
| 286827 | TRIM59 | 3q26.1 | tripartite motif-containing 59 | - | Yes | - | - |
| 347733 | TUBB2B | 6p25 | tubulin, beta 2B | - | - | Yes | - |
| 359845 | FAM101B | 17p13 | family with sequence similarity 101, member B | - | Yes | - | - |
| 388524 | LOC388524 | 19p12 | similar to Laminin receptor 1 | Yes | - | - | - |
